# Investigation of Cinnamic Acid Derivatives as Alternative Plasticizers for Improved Ductility of Polyvinyl Chloride Films

**DOI:** 10.3390/polym15214265

**Published:** 2023-10-30

**Authors:** Alejandro Barandiaran, Nestor Montanes, Lourdes Sanchez-Nacher, Rafael Balart, Miguel Angel Selles, Virginia Moreno

**Affiliations:** Technological Institute of Materials (ITM), Universitat Politècnica de València (UPV), Plaza Ferrándiz y Carbonell 1, 03801 Alcoy, Spain; albator@epsa.upv.es (A.B.); nesmonmu@upvnet.upv.es (N.M.); lsanchez@mcm.upv.es (L.S.-N.); rbalart@mcm.upv.es (R.B.); maselles@dimm.upv.es (M.A.S.)

**Keywords:** polyvinyl chloride, plasticizer, film, cinnamic acid, miscibility, mechanical properties

## Abstract

This study investigates the viability of cinnamic acid derivatives as alternative plasticizers for polyvinyl chloride (PVC) films by addressing concerns about conventional phthalate-based options that pose health and environmental risks. By theoretical modeling, this research evaluates the compatibility between various cinnamic acid-based plasticizers and the PVC matrix, which suggests their potential effectiveness. Additionally, the incorporation of these plasticizers notably enhances the tensile properties of PVC films, particularly in terms of ductility and elongation at break by surpassing the neat PVC. Moreover, cinnamic acid-based plasticizers induce a drop in the glass transition temperature and storage modulus by, thereby, enhancing flexibility and reducing brittleness in the material. Although a slight reduction in the onset degradation temperature is observed, it does not impede the industrial processing of PVC plastisols at temperatures up to 190 °C. Optically, plasticized films exhibit high transparency with minimal UV and visible light absorption, which renders them suitable for applications necessitating clarity. The water vapor transmission rate analysis indicates increased permeability, influenced by molecular volumes. Atomic force microscopy reveals a compacted, homogeneous surface structure in most plasticized films, which signifies improved film quality. Thus, utilizing cinnamic acid derivatives as PVC plasticizers offers substantial mechanical and structural benefits, while compatibility ensures effective integration by contributing to environmentally sustainable PVC formulations with enhanced performance.

## 1. Introduction

PVC is one of the materials that significantly impacts industrial and economic sectors worldwide. Its market size was valued at USD 40–60 billion in 2021, and it is projected to increase to USD 82.49 billion by 2030, mainly driven by its extensive use in the construction, automotive and consumer industries [1]. This is attributed to its diverse qualities, such as high chemical resistance, water resistance and durability against harsh weather conditions. PVC also exhibits remarkable mechanical properties, including high-impact resistance, good print adhesion, high transparency and self-extinguishing capability. Its carbon footprint is also lower compared to other materials during production, and given its life cycle, low manufacturing cost and excellent malleability [2]. However, in its pure form, PVC is brittle and prone to structural degradation at high temperatures, unlike other commodity polymers like polyethylene (PE) or polystyrene (PS). Consequently, there are certain limitations with its processing and industrial applications. Therefore, incorporating additives and process modifiers, such as plasticizers, is often necessary to minimize the material’s negative impact.

PVC can be classified as a rigid form, characterized by short and robust intermolecular forces, primarily used in the construction industry, in pipes, carpentry, and other applications. It can also be flexible, achieved by adding a plasticizer that expands the intermolecular free volume and prevents PVC molecules from coming closer to one another. Flexible PVC is commonly used to manufacture toys, shoe components, wiring, and other applications. Plasticizers can be categorized as primary or secondary. Primary plasticizers have high miscibility with PVC, typically come in a liquid form, and are responsible for significant plasticization effects. Secondary plasticizers have limited miscibility and are used to reduce costs, such as chlorinated paraffin wax (CPW) or chlorinated paraffin oils (CPOs) [3]. Given their small molecular size, primary plasticizers tend to migrate due to their high volatility [4]. Conversely, secondary plasticizers, with a higher molecular weight when combined with primary plasticizers, reduce this migration by establishing intermolecular interactions between PVC chains and plasticizers [5]. The persistence of additives incorporated into PVC or any other material plays a crucial role in the material itself due to the potential loss of properties and the environment. Plasticizer migration is an important issue to be considered for broad PVC use because some migrated plasticizers can be potentially toxic for humans or have a negative impact on environment. This is the case with phthalates, the most commonly used plasticizers for PVC. Under certain conditions, these phthalates migrate from the material because they are not chemically bonded [6], with di(2-ethylhexyl)-phthalate (DEHP) being the most popular and detected as a contaminant throughout the environment [5]. To assess the interaction of plasticizers with the PVC polymer matrix, predictive techniques, such as Hildebrand solubility parameters, polarity parameters or Flory–Huggins interaction parameters, can be employed.

The typical plasticizers used in PVC are phthalate esters, of which some notable examples are di(2-ethylhexyl) phthalate (DEHP), also known as dioctyl phthalate (DOP), diisononyl phthalate (DINP), diisodecyl phthalate (DIDP) and di(2-propylheptyl) phthalate (DPHP). These phthalates dominate the PVC plasticizer market with a 70% market share [7], although this figure is expected to significantly lower in forthcoming years. As previously mentioned, the migration of these phthalate additives poses environmental risks. Reducing the use of these conventional plasticizers is driven by the growing demand for less environmentally harmful alternatives that can be processed by conventional techniques and can compete in performance and cost terms [8,9,10,11]. Additionally, there is widespread pressure from governments, retailers and consumers to reduce the consumption of phthalates due to health risks. The European Union, through Regulation (EC) No. 1223/2009 and Directive 2005/84/EC of the European Parliament and the Council, has listed several phthalate components, namely dibutyl phthalate (DBP), DEHP, bis(2-methoxyethyl) phthalate, n-pentyl-isopentyl phthalate, di-n-pentyl phthalate, diisopentyl phthalate and butylbenzyl phthalate (BBP), as substances classified as carcinogenic, mutagenic or toxic for reproduction in cosmetic products, toys or childcare articles [12].

Current research efforts focus on identifying and utilizing biobased alternatives, including vegetable oils, epoxidized oils, sugar alcohols (i.e., glycerol and xylitol) or esters derived from natural acids, such as triethyl citrate (TEC), which is a favored plasticizer derived from the esterification of citric acid and ethanol [13]. By synthesizing 12–14 alkyl ether tributyl citrate (AETBC), Han et al. were able to obtain a plasticizer that improved the mechanical properties and migration stability performance of dioctyl phthalate (DOP), tributyl citrate (TBC) and acetyl tributyl citrate (ATBC), and also improved the thermal stability of the last two [14]. Natural plasticizers based on aliphatic, polymeric or phosphoric compounds are also being investigated [15]. According to a study conducted by Ceresana, it is estimated that 90% of global plasticizer consumption is spent on producing flexible PVC. While phthalate plasticizers remain the largest and most widely used group, phthalate-free alternatives are expected to capture a 22% market share by 2026 [16].

It has been demonstrated that natural plasticizers can enhance the performance of flexible PVC both mechanically and in migration capacity terms. These biobased alternatives offer the advantage of non-toxicity and additional benefits, such as increased light and heat stability, which lead to not only reduced degradation, but also to renewable and recyclability properties [17]. For instance, Abdelghany et al. found that PVC films manufactured with polyvinyl acetate (PVA) exhibit good antibacterial properties [18]. In another study, Jie et al. studied a novel bio-oil-based hyperbranched ester plasticizer and its effects on PVC soft films where they investigated the results of SOHE substitution of petroleum-based dioctyl phthalate (DOP) in soft PVC samples [19]. Ledniowska et al. reported the potential of esters of succinic, and oleic acid with propylene glycol. These biobased plasticizers offer similar performance to that of DEHP and diisononyl phthalate (DINP), along with a 70% reduction in overall migration and increased thermal stability [20]. The potential of these alternative and natural plasticizers is evident, and their impact on the industry is expected to continue to grow in forthcoming years. In fact it was the work of Bocqué et al. on petrochemical and bio-based plasticizers that compiled information on their plasticizing power and theoretically predicted that cinnamic acid-derived esters, such as cinnamates or coumarates, may exhibit good plasticizing properties [21].

This work explores, for the first time, the plasticizing potential of different esters of cinnamic acid on PVC, where the excellent plasticizing power of these esters on PLA has already been observed in a previous study [22]. Thus, the potential of different compounds derived from cinnamic acid as environmentally friendly plasticizers for PVC is addressed. In regards to the cinnamates, this work addresses the efficiency of methyl trans-cinnamate, ethyl cinnamate, isobutyl cinnamate, allyl cinnamate, cinnamyl isobutyrate, cinnamyl cinnamate, benzyl cinnamate, and phenethyl cinnamate.

## 2. Materials and Methods

### 2.1. Materials

**Figure 1:**
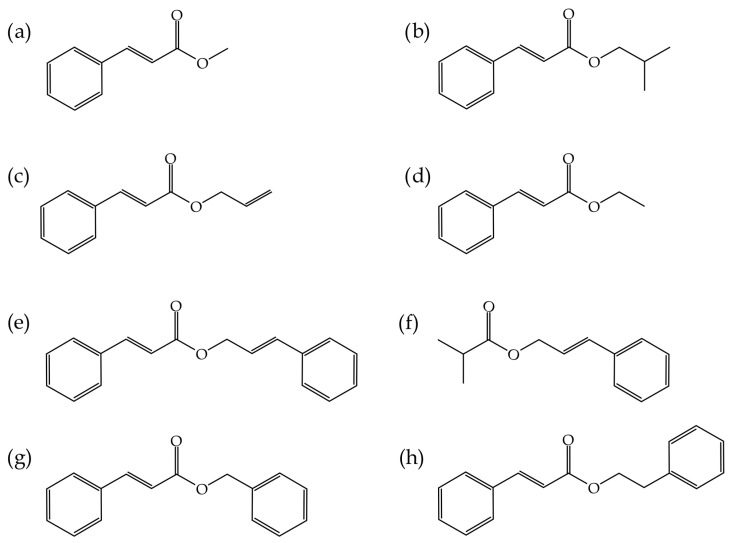
Schematic representation of the chemical structure of (**a**) methyl trans-cinnamate (MC), (**b**) isobutyl cinnamate (IC), (**c**) allyl cinnamate (AC), (**d**) ethyl cinnamate (EC), (**e**) cinnamyl cinnamate (CC), (**f**) cinnamyl isobutyrate (CI), (**g**) benzyl cinnamate (BC) and (**h**) phenethyl cinnamate (PC).

The poly(vinyl chloride)–PVC resin used in this study, manufactured according to an emulsion or microsuspension process (emulsion PVC), was commercial-grade LACOVYL^®^ PB 1172H, supplied by Kem One (Lyon, France) with a density of 1.38 g cm^−3^ and a k value of 68. Several compounds derived from cinnamic acid were provided by Sigma-Aldrich (Steinheim, Germany), including ethyl cinnamate (EC), methyl trans-cinnamate (MC), isobutyl cinnamate (IC), allyl cinnamate (AC), cinnamyl cinnamate (CC), cinnamyl isobutyrate (CI), benzyl cinnamate (BC) and phenethyl cinnamate (PC). Figure 1 illustrates a schematic representation of the chemical structure of the different cinnamates used as plasticizers for PVC, while Table 1 presents the critical physico-chemical properties of these eight cinnamic acid-derivatives.

Despite the films developed in this study being obtained by the solvent casting method, it is important for a plasticizer to have a high boiling point which prevents plasticizer loss during processing under industrial conditions (within the 160–200 °C range). These cinnamic acid derivatives were selected because of their high boiling point at 7, which is related to their molecular weight. The molar volume was calculated as the ratio of the molecular weight to density, and it plays a valuable role in obtaining the solubility parameters. Some cinnamic acid-derivatives are liquids at room temperature (i.e., IC, AC, CI), while others show relatively low melting points, which is also a key feature to use them as potential plasticizers under industrial conditions, as pointed out by Bonifacio et al. [23].

### 2.2. Theoretical Solubility Parameters

By means of different theoretical approaches, it is possible to predict the compatibility/miscibility between a polymer and additives. Several factors can influence the intermolecular entanglement of the chains in a plasticized polymer, such as the nature of the plasticizer, its volatility, migration, polarity, or employed the mixing system, which involves temperature, pressure, humidity or solvents [24]. However theoretically, the most important aspect that can be calculated and evaluated is their affinity based on solubility parameters, in this case between PVC and different types of cinnamic acid-derived plasticizers. According to Venkatram et al. [25], the Hildebrand model is usually more accurate for non-polar compounds, such as the plasticizers used in this research. The model states that for good miscibility to occur, the values of the Hildebrand solubility parameters should be as close as possible. The Hildebrand solubility parameter can be calculated as the square root of the cohesive energy density, which is the heat of vaporization divided by the molar volume. Alternatively, it can be determined using the individual parameters proposed by Hansen, known as the polar component (*δ_p_*), dispersion component (*δ_d_*) and hydrogen-bonding component (*δ_h_*). These individual parameters can be obtained by using different group contribution methods, such as the Hoftyzer and Van Krevelen or the Hoy method, which are commonly used in this context. The global Hildebrand solubility parameter (*δ*) is obtained by taking the square root of the sum of the squared individual parameters, as shown in Equation (1):

(1) $$\delta =\sqrt{{\delta_{d}}^{2}+{\delta_{p}}^{2}+{\delta_{h}}^{2}}$$

where:

(2) $$\delta_{d}=\frac{\Sigma F_{di}}{V}$$

(3) $$\delta_{p}=\frac{\sqrt{\Sigma F_{pi}^{2}}}{V}$$

(4) $$\delta_{h}=\sqrt{\frac{\Sigma E_{hi}}{V}}$$

For each chemical group, constant values are assigned to the variables of dispersion forces (*F_di_*) and polar forces (*F_pi_*), as well as the molar volume (*V*), to estimate the contributions of the dispersion (*δ_d_*) and polar (*δ_p_*) components, respectively (Equations (2) and (3)). Similarly, the cohesive energy constants for hydrogen bonding, *E_hi_*, for each structural group are well-known and, thus, enable the calculation of the contribution of the hydrogen-bonding component (*δ_h_*) (Equation (4)).

### 2.3. Processing Plasticized PLA Formulations

Films were manufactured following the solvent casting method proposed by Patil et al. [26], with some modifications. In this case, the procedure involved preparing a solution for each film, which consisted of 800 mg of PVC powder + 400 mg of cinnamate (50 parts per hundred) in 18 mL of tetrahydrofuran (THF). Subsequently, solutions were stirred at 600 rpm for approximately 30 min until complete homogenization was achieved. Finally, the solution was poured into a Petri glass dish (9 cm in diameter) to cover all the Petri surface, and the solvent was allowed to evaporate at room temperature for 72 h. Drying at higher temperatures was omitted because the low molecular weight of plasticizers could lead to some plasticizer loss at high temperatures. Each plasticized PVC formulation was prepared in quintuplicate. Once films were obtained, they were removed from Petri dishes and stored between aluminum foils to prevent any form of degradation, such as photodegradation, because PVC is sensitive to light due to its relatively low dissociation energies for its molecular groups, or hydrolysis of the ester groups contained in cinnamic acid-based plasticizers [27]. PVC films containing cinnamic acid-based plasticizers are lighter and stable because PVC is light-sensitive. These types of compounds offer functional groups that bind strongly to PVC films, which make them stronger and, therefore, stabler in both thermal and mechanical terms.

### 2.4. Characterization of Plasticized PVC Formulations

#### 2.4.1. Mechanical Properties

The mechanical properties of the unplasticized PVC and PVC formulations plasticized with cinnamic acid-based plasticizers were determined by tensile tests. The different film samples were tested using a universal testing machine ELIB 30 from S.A.E. Ibertest (Madrid, Spain), equipped with pneumatic grips designed explicitly for film testing. Tests followed Standard ISO 527 [28] on sample preparation and test conditions. A sufficient number of samples from each formulation was analyzed to obtain five reliable results for the main tensile parameters, based on the average of these samples. Tests were performed using a 100 N load cell with a 5 mm min^−1^ crosshead speed.

#### 2.4.2. Thermal Properties

The thermal stability and degradation of the PVC and plasticized PVC formulations were evaluated using thermogravimetric analysis (TGA) on a Mettler-Toledo TGA/SDTA 851 thermobalance (Schwerzenbach, Switzerland). All the analyses were performed in a nitrogen atmosphere on the samples weighing approximately 5 mg, with a temperature cycle ranging from 30 to 700 °C at a constant heating rate of 20 °C min^−1^.

#### 2.4.3. Thermomechanical Test

The thermomechanical properties were evaluated using the dynamic mechanical thermal analysis (DMTA) technique on a DMA-1 model from Mettler-Toledo S.A. (Barcelona, Spain). Tests were carried out on rectangular film specimens measuring 10 × 5 mm^2^. The temperature cycle applied to samples ranged from −100 to 100 °C at a constant heating rate of 2 °C min^−1^. The offset strength was set at 1 N, the offset deformation at 150% and the control deformation at 6 μm.

#### 2.4.4. Fourier Transform Infrared Spectroscopy

Films were analyzed using attenuated total reflectance Fourier transform infrared spectroscopy (ATR-FTIR) on a Perkin-Elmer BX infrared spectrometer from Perkin Elmer Spain, S.L. (Madrid, Spain). Before sampling, a background measurement was taken in the wavenumber region between 4000–600 cm^−1^ to compensate for the environmental effects of moisture and carbon dioxide. The FTIR-ATR spectra of the unplasticized PVC and plasticized PVC with the cinnamic acid-based plasticizers were obtained as the average scan of 64 scans at a resolution of 4 cm^−1^.

#### 2.4.5. UV-Visible Spectrophotometry

UV-Visible spectrophotometry (UV-Vis) was used to evaluate the shielding properties of films against ultraviolet (UV) light (200–400 nm), high-energy visible blue light (HEV, 400–450 nm) and visible light (Vis) (400–800 nm) [29]. The neat PVC and plasticized PVC film samples were analyzed at room temperature within the 200–800 nm absorption spectrum by a UV-Vis spectrophotometer Perkin Elmer Lambda 35 (Waltham, MA, USA).

#### 2.4.6. Water Vapor Transmission Rate

According to the E96/E96M-16 standard [30], the water vapor permeability of the unplasticized PVC and plasticized PVC formulations with the cinnamic acid-based plasticizers was evaluated. The films from each sample were placed in cups containing 2 g of silica gel, which had been pre-dried at 200 °C for 24 h. Once cups were sealed with films, they were placed in a desiccator programmed to maintain 90% relative humidity (RH) at 23 °C. Each sample was weighed hourly for 7 h and each formulation was analyzed in triplicate. The water vapor transmission rate (WVTR) was calculated according to Equation (5):

(5) $$WVTR=\frac{\frac{G}{t}\cdot l}{A\cdot S\cdot (R_{1}-R_{2})}$$

where *G*/*t* represents the slope of the straight line (g/h); *l* is film thickness (μm); *A* is the tested area (cup mouth area, m^2^); *S* is the saturation vapor pressure at the test temperature (mm Hg); Δ*R* is the relative humidity difference between the source and the vapor sink expressed as a fraction.

#### 2.4.7. Atomic Force Microscopy

Atomic force microscopy (AFM) was employed for the sample characterization using a Multimode 8 with Nanoscope V Controller (Bruker, Billerica, MA, USA) with an integrated silicon tip/cantilever. The AFM height images were obtained after operating in the tapping mode and the diameters of samples were calculated from the height profiles.

#### 2.4.8. Statistical Analysis

To measure the significant differences among samples, they were evaluated at the 95% confidence level (*p* ≤ 0.05) by a one-way analysis of variance (ANOVA) following a Tukey’s test. The software employed for this propose was the open-source R software v 4.2.3 (http://www.r-project.org), date accessed: 21 October 2023.

## 3. Results and Discussion

### 3.1. Theoretical Approach to Miscibility of PLA and Cinnamates

Table 2 presents the three solubility component values described by Hansen, which were obtained by the group contribution method proposed by Hoftyzer and Van Krevelen, as well as the Hildebrand solubility parameter (δ). The difference between the solubility parameters of pure PVC and the proposed potential plasticizers (Δδ) appears in Table 2. The solubility parameter contributions proposed by Hansen were obtained from [31,32] and the average of these values was calculated to determine the Hildebrand solubility parameter of pure PVC, which was 20.68 MPa^1/2^.

According to the results, a general theoretical estimate can be made of the miscibility between PVC and the proposed plasticizers. The values of the difference column between the Hildebrand solubility parameter and that of pure PVC varied between 0.03 and 2.00 MPa^1/2^. Allyl cinnamate was expected to exhibit the highest compatibility, while benzyl cinnamate would show the lowest. It is important to highlight that the solubility parameter values of almost all the proposed plasticizers derived from cinnamic acid were sufficiently close, which indicates that all the proposed plasticizers would generally exhibit good miscibility.

### 3.2. Analysis of Thermal Stability and Residual Plasticizer after Processing

Manufacturing processes, temperature, humidity and other external agents can degrade materials and cause them to lose their initial properties. Similar phenomena can occur with plasticizers because their ability to migrate from the polymer matrix depends on their nature; for example, whether they are monomeric, polymeric or oligomeric, as well as the degree of branching in monomeric chains. To minimize material degradation in this regard, employing highly branched polymeric or oligomeric plasticizers is recommended [33]. PVC has a good affinity for esters, and increasing the molecular weight of plasticizers enhances the interactions between the carbonyl group of esters and the CHCl groups of PVC, which results in improved tensile strength of the material. Conversely, using low-molecular-weight additives leads to materials with easier processability and greater elongation [10]. However, molecular weight directly affects loss of volatiles and migration toward liquids and solid absorbents. With low-molecular-weight additives, this phenomenon is pronounced due to the bigger free volume that they provide, which, thus, facilitates diffusion from the material to the surface and subsequent evaporation into the environment [5].

**Figure 2:**
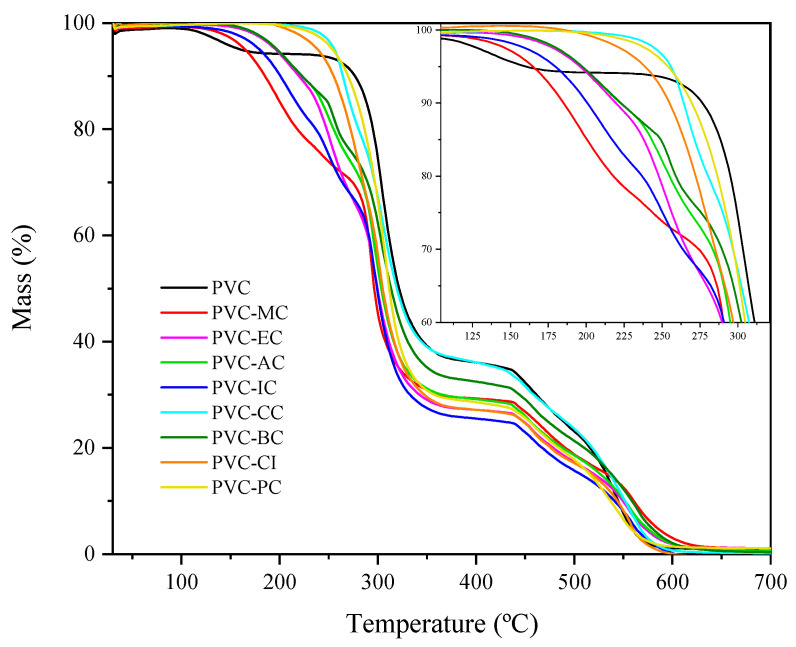
TGA graph of mass % vs. temperature of the pure PVC, methyl trans-cinnamate (MC), isobutyl cinnamate (IC), allyl cinnamate (AC), ethyl cinnamate (EC), cinnamyl cinnamate (CC), cinnamyl isobutyrate (CI), benzyl cinnamate (BC) and phenethyl cinnamate (PC) within the 30 to 700 °C range.

According to Figure 2, pure PVC exhibits the typical graph for this material [34]. The mass loss observed around 109 °C was attributed to the residual solvent (THF) retained by the PVC molecules and must not be considered for calculating the main thermal degradation parameters. We can clearly see two behaviors with regard to thermal stability. The low-molecular-weight cinnamic acid-based plasticizers showed lower thermal stability compared to the high-molecular-weight cinnamic acid-based plasticizers. Hence, the plasticizers with higher volatility are MC, IC, EC and AC with T_5_ values ranging from 165.54 °C (MC) to 197.52 °C (AC). In contrast, the plasticizers derived from cinnamic acid show less tendency to volatilization as their T_5_ values suggest, which ranged from 238.93 (CI) to 217.17 (CC).

These results are particularly interesting because one of the significant concerns about PVC and phthalates is their migration into the environment under certain conditions, which poses health risks [35]. The theoretical miscibility results support the existence of good compatibility between the used PVC and the cinnamic acid-based plasticizers. To further analyze the thermal stability of compounds, Table 3 presents the values of temperatures T5, T15 and T50 corresponding to the 5%, 15% and 50% mass losses, respectively, which are representative of the thermal degradation of PVC and its plasticized formulations with the cinnamic acid-based plasticizers. By taking into account that industrially plasticized PVC is processed within the temperature range between 160 °C and 200 °C, less plasticizer loss is expected in all the plasticized PVC formulations containing the cinnamic acid-based plasticizers. In general, the higher the molecular weight of the monomeric plasticizer, the better thermal stability is. According to Shi et al. [31], this can be attributed to the increased presence of ester bonds and hydroxyl and benzene groups, which have good thermal stability. The low stability of PVC, where the first decomposition occurs at 150 °C, is attributed to the presence of defective segments in polymer chains [36].

### 3.3. Mechanical and Thermomechanical Properties of the Plasticized PVC with the Cinnamic Acid-Based Plasticizers

Analyzing the mechanical properties of PVC films is one way to assess the effectiveness of plasticizers. The results of the tensile strength tests are presented in Table 4. As observed, all the cinnamate formulations at 50 parts per hundred exhibit significantly improved ductility compared to the unplasticized PVC. This is consistent with the findings from the theoretical solubility parameter study of PVC-cinnamic acid-based plasticizers. As expected, the plasticized PVC formulations were characterized by lower tensile strength than the unplasticized PVC, which ranged from 9.6 to 22.2 MPa, and was much lower in elastic modulus terms, with values ranging from 6 to 120 MPa. The unusual behavior of cinnamyl cinnamate (CC) in terms of its elastic modulus compared to the other cinnamic acid-based plasticizers is noteworthy because it significantly outperformed them without sacrificing elastic and strength properties. It exhibited the highest tensile strength with a value of 22.2 MPa, which was not far from that of the unplasticized PVC, and it also offered comparable elongation to the other cinnamic acid-based plasticizers. For elongation at break, very high values were obtained compared to the unplasticized PVC, which ranged above 260% and reached nearly 400% for CI with a 372% elongation. This corroborated the exceptional plasticization efficiency that these cinnamic acid-based plasticizers confer PVC. These values are comparable to those obtained by Feng et al. [37], who used DOP and other kitchen waste-based transesterified and epoxidized plasticizers at a concentration of 40 parts per hundred. Their elongation values ranged from 376% to 422%.

The purpose of plasticization, apart from modifying mechanical properties toward more ductile materials, is to achieve a compound with a lower glass transition temperature (T_g_) than the polymer itself. Below its characteristic T_g_, a polymer exhibits rigid, brittle behavior in a glassy state, where molecular chains motion is very restricted. Chains have greater mobility above T_g_ and, thus, leads to increased ductility [38]. The effect of plasticization is to increase the free volume of the base polymer by making the distance between molecular chains longer to improve overall mobility.

**Figure 3:**
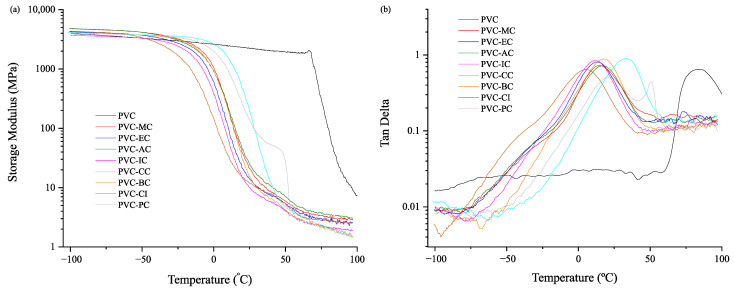
Plot evolution of (**a**) the storage modulus (E’) vs. temperature and (**b**) the dynamic damping factor (tan d) vs. temperature for the unplasticized PVC and plasticized PVC with the cinnamic acid-based plasticizers within the −100 to 100 °C range.

The PVC grade used in this study had a T_g_ of 83.8 °C. The formulations with plasticizers exhibited lower T_g_ values and storage modulus. Depending on their molecular structure, these values could vary to a greater or lesser extent. Figure 3 shows that the eight cinnamic acid-based plasticizers used in this research present a range of T_g_ values from 5.0 °C to 33.4 °C, which is a key factor in assessing the plasticization efficiency of the selected plasticizers. Consistently with the results shown in Table 4 for the mechanical tests, the plasticizers that provided the greatest elongation, namely PVC-CI (372%), PVC-MC (343%), PVC-EC (312%), and PVC-IC (308%), also achieved the most significant reduction in T_g_, with values of 5.0 °C, 14.7 °C, 12.9 °C and 11.7 °C, respectively, as indicated in Table 5. Those plasticizers with elongation at break below 300% in the mechanical tests, namely BC (261.4%), CC (279.3%), and PC (298.1%), exhibited the highest glass transition temperatures of 17.5 °C, 33.4 °C and 21.2 °C, respectively. In any case, the results obtained with the cinnamic acid-based plasticizers are very interesting and reveal an exceptional plasticizing effect that resulted from the increased free volume and reduced molecular interactions, which favored greater chain mobility at lower temperatures [22].

Pure PVC exhibits a glassy state up to ≈70 °C. Beyond that temperature and above the T_g_ of 83.8 °C, the storage modulus decreases substantially (by three orders of magnitude). Significantly lower storage moduli were observed for the plasticized formulations. Table 5 reports the storage modulus values at 30 °C and 70 °C, which ranged between 7.8–84.6 MPa and 2.3–3.9 MPa, respectively, and are typical of the rubbery state above the glass transition temperature.

### 3.4. Optical Properties and Spectroscopic Characterization

**Figure 4:**
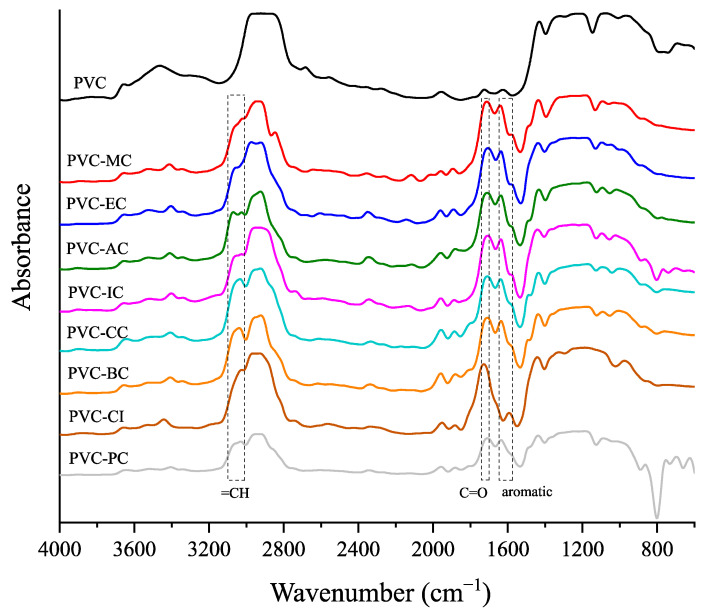
FTIR spectra of the neat PVC and plasticized PVC formulations with the different cinnamic acid-based plasticizers within the 4000 to 800 cm^−1^ range.

The infrared spectra of the unplasticized PVC and the plasticized PVC formulations containing the cinnamic acid-based plasticizers are shown in Figure 4. According to the literature [39], the infrared peaks associated with PVC include CH stretching within the range of 2950–2850 cm^−1^, CH2 bond deformation at 1349 cm^−1^, out-of-plane CH rocking within the range of 1220–1190 cm^−1^, out-of-plane trans-CH wagging at approximately 950 cm^−1^ and a C-Cl group with a peak band located at 834–805 cm^−1^. In addition to the known peaks of neat PVC, a characteristic peak of the cinnamic-acid based compounds can be observed within the wavenumber range of 3100–3010 cm^−1^, which is attributed to =CH [40]. The absorption peaks of the carbonyl and aromatic groups, which range between 1700–1741 cm^−1^ and 1647–1572 cm^−1^ [41], respectively, are characteristic of the cinnamic acid-based plasticizers. They are not present in neat PVC, but appear in the plasticized PVC formulations with the different cinnamic acid-based plasticizers. This clearly evidences successful plasticization.

**Figure 5:**
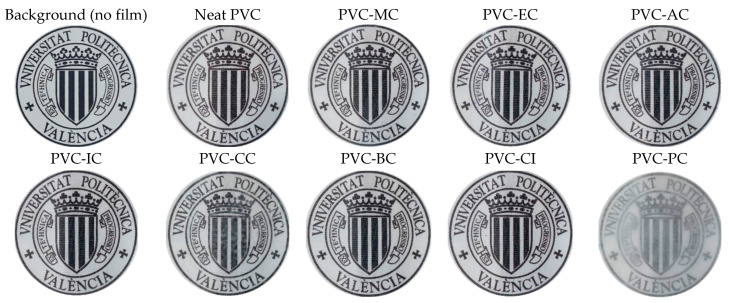
Optical images showing the transparency of films of the neat PVC and plasticized PVC films with the different cinnamic acid-based esters.

**Figure 6:**
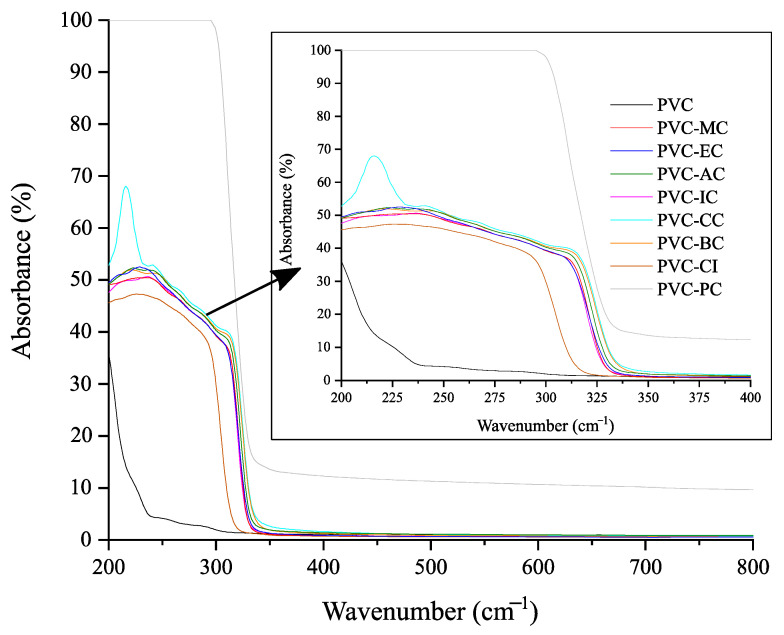
UV-Vis spectra of the neat PVC and plasticized PVC formulations with the different cinnamic acid-based plasticizers within the 200–800 nm range.

**Figure 7:**
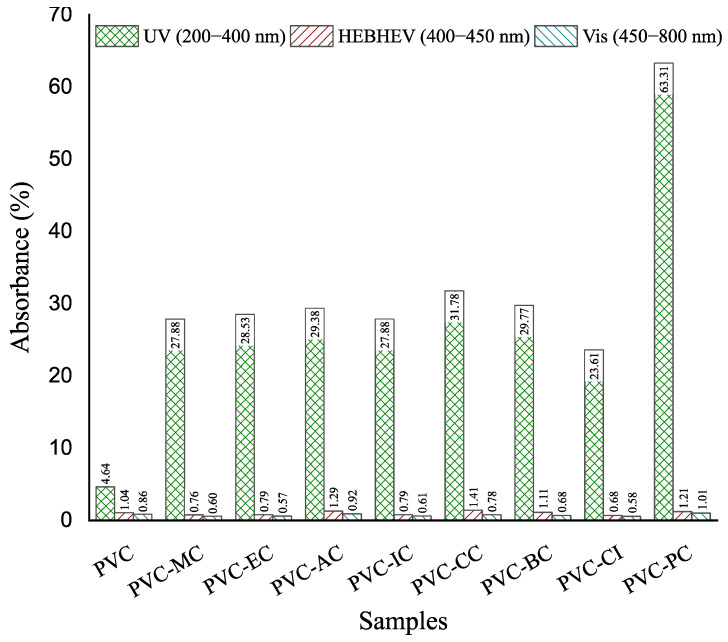
Percentage absorption of ultraviolet (UV), high energy visible light (HEV) and visible light (Vis) of the films of the neat PVC and plasticized PVC formulations with the different cinnamic acid-based plasticizers within the 200–800 nm range.

Regarding transparency, Figure 5 shows images of the films obtained with the unplasticized PVC and plasticized formulations with the different cinnamic acid-based plasticizers. Once the films with the corresponding additives were obtained by the solvent casting method, materials were placed on top of the university logo for its optical properties. None of the films underwent any detectable color change, and only the films plasticized with phenethyl cinnamate (PC) and cinnamyl cinnamate (CC) showed decreased transparency, with PC exhibiting the most significant reduction. As shown, films generally exhibited relatively low UV radiation absorption levels, except for the plasticized PVC film containing phenethyl cinnamate. Regarding high-energy blue (HEB), which comprises particularly intense light waves that are emitted by many modern electronics and even compact fluorescent light bulbs, and visible light, all the films demonstrated high transmittance levels of 99%, as observed in Figure 6 and Figure 7. To ensure good protection against potentially harmful UV and HEB radiation for human optical health, incorporating additional UV-absorbing additives like benzotriazole or benzophenone, along with pigments for HEB, would be necessary [42]. Further research into the compatibility between the cinnamic acid-based plasticizers and these compounds would be valuable. However, in the initial assessments, it was notable that the cinnamic acid-based plasticizers provided outstanding plasticization properties to PVC while preserving its transparency. These results are consistent with the results obtained for optical and tensile properties, and indicate that the samples containing cinnamic acid-based plasticizers had a significant effect on flexibility properties and exhibited the best toughness compared to commercial dioctyl terephthalate (DOTP) and diisononyl phthalate (DINP) [34].

According to the bibliography [5], in which the mechanical properties, glass transition temperatures and the results of the different migration tests are collected, a comparison can be made of the results obtained with the most widely used commercial plasticizers in PVC films. As seen, all the collected data were compared to the “standard mixture”, and also to the mixtures with a common commercial plasticizer, which is usually diethylhexyl phthalate (DEHP) or DOP.

### 3.5. Water Vapor Transmission Rate (WVTR) and Surface Morphology

According to the values presented in Table 6, the unplasticized PVC exhibited the highest resistance to water vapor transmission. This was attributed to the rigid and resistant nature of the material’s polymer structure. Upon adding plasticizers, these values increased within the 1.11 × 10^−12^ g µm m^2^ h^−1^ Pa^−1^ to 4.18 × 10^−12^ g µm m^2^ h^−1^ Pa^−1^ range, with PVC-CI showing the highest permeability and PVC-PC exhibiting the lowest. The increase in water vapor transmission was attributed to the increase in the free volume in the plasticized PVC formulations with the cinnamic acid-based plasticizers, which allowed water molecules to travel through the material more easily. The plasticizers with larger molecular volumes displayed lower permeability. This has been demonstrated to be due to the increased interaction of hydrogen bonds, which results in the immobilization of water molecules, and consequently reduces the permeability of films [24]. Table 6 also includes the significant difference among the samples according to the standard mentioned in the experimental section.

**Figure 8:**
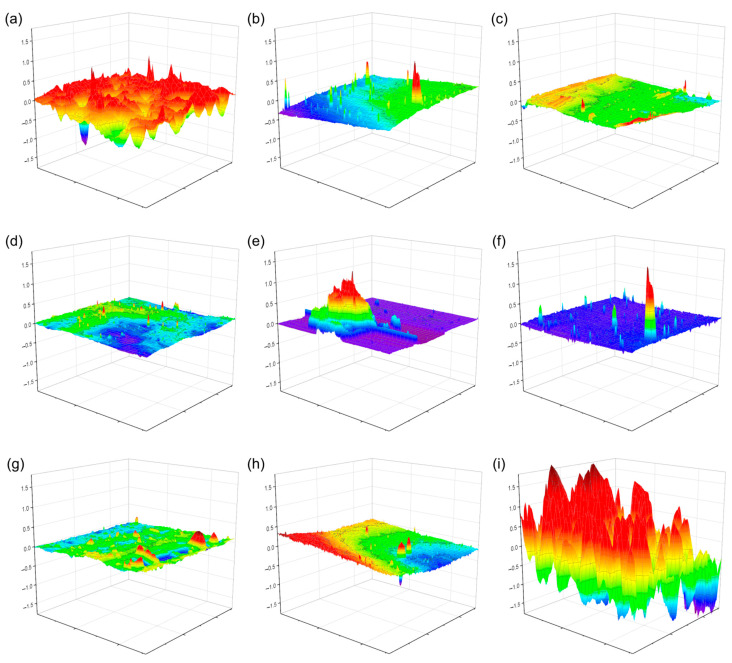
AFM topographic images of the films of the neat PVC and plasticized PVC formulations with the different cinnamic acid-based plasticizers: (**a**) pure PVC, (**b**) methyl trans-cinnamate (MC), (**c**) isobutyl cinnamate (IC), (**d**) allyl cinnamate (AC), (**e**) ethyl cinnamate (EC), (**f**) cinnamyl cinnamate (CC), (**g**) cinnamyl isobutyrate (CI), (**h**) benzyl cinnamate (BC) and (**i**) phenethyl cinnamate (PC). Red color corresponds to the maximum value and purple the minimum value.

Atomic force microscopy (AFM) was employed to examine the surface topography of the plasticized PVC films with the different cinnamic acid-based plasticizers. The AFM topographic images (see Figure 8) generally showed homogeneity in the observed film fragment’s surface roughness and a smoothed compact structure. However, with PVC-PC (plasticized with phenethyl cinnamate), a much more irregular surface was obtained, possibly due to the material’s photodegradation because it exhibited higher UV absorption capacity [43], as shown by the UV-Vis spectroscopy study. Additionally, cavities were observed for the neat PVC film, likely caused by the volatilization of the solvent and resulting in bubble-like formations on the surface. With the plasticized PVC films, these formations were barely noticeable because the plasticizer compacted the structure and prevented this phenomenon by hindering solvent release. Any released solvent generates much smaller occlusions and gives the impression of a smoothed surface. It has been shown that the roughness factor (Rq) decreases in the presence of agents that plasticize the material when irradiated [44,45].

Finally, Table 7 summarizes the advantages, the type of plasticizer used in the method and the processing of several studies based on the plasticization of PVC with different types of plasticizers.

## 4. Conclusions

This work investigated the compatibility and properties of plasticized PVC formulations with various cinnamic acid-based compounds as potential plasticizers. Plasticizers exhibited good compatibility with PVC based on their Hildebrand solubility parameters, with a difference ranging from 0.03 to 2.00 MPa^1/2^. While the PVC films with the cinnamic acid-based plasticizers showed lesser thermal stability compared to the neat PVC, most plasticizers had minimal volatilization as shown by the TGA. This volatilization was directly linked with the molecular weight of the cinnamic acid-based compound. Notwithstanding, the thermal stability of these environmentally friendly plasticizers provided to PVC does not compromise either processing conditions or typical work temperatures because the lowered thermal stability was observed only at high temperatures, as the TGA revealed.

The addition of the cinnamic acid-based plasticizers notably improved the ductility of PVC films. Tensile strength values ranged from 9.6 to 22.2 MPa, and the elastic modulus values ranged from 6 to 120 MPa. Cinnamyl cinnamate (CC) exhibited exceptional behavior with a tensile strength of 22.22 MPa, akin to the neat PVC, while maintaining great elongation at break. The elongation at break for the plasticized PVC formulations surpassed 260% for all the selected cinnamic acid-based compounds, which highlights their potential as effective plasticizers for enhancing PVC film properties. The dynamic mechanical-thermal analysis revealed that plasticized PVC formulations with cinnamic acid-based compounds exhibited lower T_g_ compared to the neat PVC. The T_g_ of the neat PVC at 83.8 °C was notably lowered with these plasticizers and ranged from 5 °C to 33.4 °C, which confirms their exceptional plasticization efficiency.

The plasticized PVC films with the cinnamic acid-based compounds demonstrated excellent transparency and relatively low UV radiation absorption levels, except for the phenethyl cinnamate (PC) plasticizer, which displayed reduced transparency. The water vapor transmission rate analysis indicated increased permeability to water vapor with the addition of these plasticizers, which fell in line with expectations.

In summary, cinnamic acid derivatives offer a promising alternative to conventional plasticizers in PVC formulations by providing improved ductile properties and efficient plasticization.

## Figures and Tables

**Table 1:** Physico-chemical and thermal properties of: (a) methyl trans-cinnamate (MC); (b) isobutyl cinnamate (IC); (c) allyl cinnamate (AC), (d) ethyl cinnamate (EC); (e) cinnamyl cinnamate (CC); (f) cinnamyl isobutyrate (CI); (g) benzyl cinnamate (BC) and (h) phenethyl cinnamate (PC).

Cinnamic Acid-Derivative	Melting Point (°C)	Boiling Point (°C)	Molecular Weight (g mol^−1^)	Density (g cm^−3^)	Molar Volume (cm^3^ mol^−1^)
MC	34–38 ^a^	260–262 ^a^	162.188	1.092	148.524
IC	-	287 ^a^	204.269	1.005	203.658
AC	-	289 ^a^	188.226	1.053	178.752
EC	6–7 ^a^	271 ^a^	176.215	1.050	168.305
CC	42–45 ^a^	370 ^a^	264.324	1.121	235.793
CI	-	295–297 ^a^	204.269	1.008	202.648
BC	37–39 ^a^	195–200 ^b^	238.286	1.11	214.672
PC	54–56 ^a^	300–301 ^a^	252.313	1.108	227.719

^a^ at 760 mm Hg/ ^b^ at 5 mm Hg.

**Table 2:** Solubility component values described by Hansen, the Hildebrand solubility parameter values and the difference between the pure PVC and the proposed potential plasticizers.

Code	$\delta_{d}$ (MPa^1/2^)	$\delta_{p}$ (MPa^1/2^)	$\delta_{h}$ (MPa^1/2^)	δ (MPa^1/2^)	Δδ (MPa^1/2^)
PVC [31,32]	18.54	8.56	3.31	20.68	-
MC	15.83	4.66	11.81	20.29	0.39
EC	16.06	6.63	10.50	20.94	0.26
AC	15.06	6.72	10.09	20.65	0.03
IC	16.47	5.92	8.43	19.58	1.10
CC	15.11	5.47	8.75	19.13	1.55
BC	16.76	7.15	11.53	22.68	2.00
CI	15.85	5.72	9.05	19.89	0.79
PC	16.91	6.59	10.63	21.95	1.27

**Table 3:** Temperatures T_5_, T_15_ and T_50_ corresponding to the 5%, 15% and 50% mass losses achieved by thermogravimetry on the unplasticized PVC and plasticized PVC with the cinnamic acid-based plasticizers.

Code	T_5_ (°C)	T_15_ (°C)	T_50_ (°C)
PVC	283.4 ± 2.7	287.4 ± 3.2	321.7 ± 3.6
PVC-MC	165.5 ± 2.1	200.3 ± 2.7	296.9 ± 2.7
PVC-EC	195.7 ± 1.9	238.2 ± 2.4	299.5 ± 2.0
PVC-AC	197.5 ± 1.7	242.7 ± 1.9	303.7 ± 2.4
PVC-IC	180.1 ± 1.9	217.3 ± 2.0	299.5 ± 2.2
PVC-CC	256.2 ± 3.7	271.4 ± 2.9	319.3 ± 1.7
PVC-BC	197.5 ± 2.4	249.2 ± 1.7	314.5 ± 1.9
PVC-CI	238.9 ± 3.3	264.2 ± 2.2	304.8 ± 2.5
PVC-PC	254.0 ± 0.7	279.3 ± 3.3	311.6 ± 1.8

**Table 4:** Results of the tensile tests of the unplasticized PVC and plasticized PVC formulations containing the different cinnamic acid-based plasticizers.

	Tensile Strength (MPa)	Young’s Modulus (MPa)	Elongation at Break (%)
PVC	37.7 ± 2.0	1023.6 ± 56.0	8.8 ± 2.0
PVC-MC	11.6 ± 0.9	7.9 ± 1.5	343.0 ± 27.3
PVC-EC	9.6 ± 0.6	5.8 ± 1.0	312.1 ± 23.4
PVC-AC	11.6 ± 1.0	7.5 ± 0.3	308.3 ± 18.0
PVC-IC	10.4 ± 0.6	8.1 ± 1.3	308.8 ± 13.5
PVC-CC	22.2 ± 1.0	119.9 ± 16.5	279.3 ± 10.2
PVC-BC	16.3 ± 1.3	17.0 ± 1.8	261.4 ± 15.9
PVC-CI	13.5 ± 0.8	6.2 ± 0.5	371.6 ± 12.8
PVC-PC	16.8 ± 0.7	47.8 ± 4.5	298.2 ± 14.4

**Table 5:** Storage modulus values at 30 °C and 70 °C obtained by dynamic thermos-mechanical analysis (DMTA) for the unplasticized PVC and plasticized PVC formulations with the different cinnamic acid-based plasticizers.

Code	Storage Modulus, E’ (MPa)		T_g_ (°C)
	at 30 °C	at 70 °C	
PVC	2008.0 ± 39.0	1077.1 ± 26.8	83.8 ± 2.4
PVC-MC	14.1 ± 1.4	3.6 ± 0.2	14.7 ± 0.5
PVC-EC	10.5 ± 0.7	3.2 ± 0.2	12.9 ± 0.4
PVC-AC	16.4 ± 1.1	3.9 ± 0.3	17.2 ± 0.3
PVC-IC	7.8 ± 0.7	2.4 ± 0.3	11.7 ± 0.4
PVC-CC	75.9 ± 2.2	2.6 ± 0.2	33.4 ± 0.8
PVC-BC	10.3 ± 0.4	2.4 ± 0.1	17.5 ± 0.4
PVC-CI	10.0 ± 0.6	2.9 ± 0.2	5.0 ± 0.3
PVC-PC	84.6 ± 2.0	2.3 ± 0.2	21.2 ± 0.7

**Table 6:** Water vapor transmission rate (WVTR) of the films of the neat PVC and plasticized PVC formulations with the different cinnamic acid-based plasticizers measured at 23 °C and relative humidity of 90%.

Code	WVTR (×10^−12^ g µm m^2^ h^−1^ Pa^−1^)
PVC	1.04 ± 0.05 ^a^
PVC-MC	1.58 ± 0.09 ^b^
PVC-EC	2.00 ± 0.12 ^b^
PVC-AC	2.92 ± 0.10 ^b^
PVC-IC	2.26 ± 0.09 ^b^
PVC-CC	1.31 ± 0.06 ^b^
PVC-BC	1.35 ± 0.05 ^b^
PVC-CI	4.18 ± 0.15 ^b^
PVC-PC	1.11 ± 0.03 ^a^

^a,b^ Different letters in the same column indicate a significant difference among samples (*p* < 0.05).

**Table 7:** Comparison of various methods of plasticizing PVC films, showing the used plasticizer, the processing and general conclusions of the obtained results.

Plastification of PVC Films with Different Plasticizers and Involved Methods			
Plasticizer(s)	Processing	Comments	Reference
2,5-furandicarboxylic acid and butyl oligoglycol ethers	Esterification synthesis	Reduction of the melt-point. Increased plasticization efficiency and drop in T_g_	[8]
Cardanol and quaternary ammonium phosphotungstate	Casting method	Improved thermal stability and drop in T_g_	[10]
Esters/ethers derived from tartaric acid	Casting method	Good plasticizing effect, displays low migration potential, and does not negatively impact the thermal stability of polymers	[46]
Hyperbranched poly(ε-caprolactone	Copolymerization	High plasticization efficiency and minimal plasticizers migration, even under very harsh conditions	[33]
Polyethylene glycol methyl ether and dimer acid	Hydrolysis of fatty acids from soybean oil at 70 °C and subsequent Diels-Alder reaction at 250 °C	Tensile properties, transparency and thermal stability of plasticized PVC increased significantly with more oxyethyl units	[11]
Hyperbranched ester plasticizer (SOHE)	Synthesis with a large number of steps	Greater thermal stability and flexibility. The migration stability of PVC samples enhances when increasing the amount of SOHE	[19]
Castor oil methyl ester, cardanol, and triethyl citrate with rosin-based plasticizers	Synthesis with a large number of steps according to free volume theory and lubricity theory	Good plasticizing efficiency and miscibility	[47]
Cardanol, cardanol acetate and epoxidized cardanol acetate	Synthesis with several steps	Overall superior flexibility, compatibility, thermal stability and workability than commercial phthalate plasticizers	[34]
Cinnamic acid derivatives	Solvent casting method	Good compatibility with PVC. Improved ductility, thermal and mechanical properties. Increased permeability	This paper

## Data Availability

The data presented in this study are available on request from the corresponding author.
